# Supplementary material for: Cryo-EM structures of a pentameric ligand-gated ion channel in liposomes
Source: eLife. 2025 Jul 16;14:RP106728. doi: 10.7554/eLife.106728 (PMC12266721; doi:10.7554/eLife.106728)
Supplement: Supplementary file 1. [file elife-106728-supp1.docx]

|  | ELIC state 1 with propylamine facing ECD outwards in liposomes with 2:1:1 POPC:POPE:POPG  EMD-49390  PDB 9NGQ | ELIC state 2 with propylamine facing ECD outwards in liposomes with 2:1:1 POPC:POPE:POPG  EMD-49391  PDB 9NGR | ELIC with propylamine facing ECD inwards in liposomes with 2:1:1 POPC:POPE:POPG  EMD-49392  PDB 9NGS | ELIC facing ECD outwards in liposomes with 2:1:1 POPC:POPE:POPG  EMD-49383  PDB 9NGF | ELIC facing ECD inwards in liposomes with 2:1:1 POPC:POPE:POPG  EMD-49384  PDB 9NGG |
| --- | --- | --- | --- | --- | --- |
| **Data collection and processing** | | | | |  |
| Magnification | 75000 | 75000 | 75000 | 75000 | 75000 |
| Voltage (kV) | 300 | 300 | 300 | 300 | 300 |
| Electron exposure (e-/ Å^2^) | 54.4 | 54.4 | 54.4 | 55.4 | 55.4 |
| Defocus range (µm) | -1 to -2.4 | -1 to -2.4 | -1 to -2.4 | -1 to -2.4 | -1 to -2.4 |
| Pixel size (Å) | 0.865 | 0.865 | 0.865 | 0.868 | 0.868 |
| Symmetry imposed | C5 | C5 | C5 | C5 | C5 |
| Initial particle images (no) | 635,388 | 635,388 | 635,388 | 570,264 | 570,264 |
| Final particle images (no) | 15,718 | 15,718 | 8,145 | 26,273 | 13,614 |
| Map resolution (Å) | 3.8 | 3.8 | 4.2 | 3.4 | 3.6 |
| FSC threshold | 0.143 | 0.143 | 0.143 | 0.143 | 0.143 |
| **Refinement** | | | | |  |
| Initial model used | PDB 8F34 | PDB 8F34 | PDB 8F34 | PDB 8F35 | PDB 8F35 |
| Model resolution (Å) | 3.8 | 3.8 | 4.2 | 3.5 | 3.7 |
| FSC threshold | 0.5 | 0.5 | 0.5 | 0.5 | 0.5 |
| Map sharpening B factor (Å^2^) | -130.301 | -130.301 | -138.788 | -117.585 | -130.345 |
| **Model composition** | | | | |  |
| Non-hydrogen atoms | 12551 | 12551 | 12549 | 12585 | 12617 |
| Protein Residues | 1535 | 1535 | 1535 | 1535 | 1535 |
| Ligands | 5 | 5 | 5 | 0 | 0 |
| **B factors (Å^2^) (0.5)** | | | | |  |
| Protein | 13.55 | 13.55 | 29.12 | 27.02 | 25.93 |
| Ligand | 9.82 | 9.82 | 9.57 | --------- | --------- |
| **R.m.s. deviations** | | | | |  |
| Bond lengths (Å) | 0.006 | 0.007 | 0.006 | 0.005 | 0.004 |
| Bond angles (°) | 1.099 | 1.132 | 1.167 | 1.083 | 1.012 |
| **Validation** | | | | |  |
| MolProbity Score | 1.77 | 1.77 | 1.53 | 1.75 | 1.61 |
| Clashscore | 8.20 | 7.92 | 4 | 7 | 4.54 |
| Poor rotamers (%) | 0 | 0 | 0 | 0 | 0 |
| **Ramachandran plot** | | | | |  |
| Favored (%) | 95.34 | 95.08 | 95.08 | 94.43 | 94.43 |
| Allowed (%) | 4.66 | 4.92 | 4.92 | 5.57 | 5.57 |
| Disallowed (%) | 0 | 0 | 0 | 0 | 0 |

|  | ELIC5 with propylamine facing ECD outwards in liposomes with 2:1:1 POPC:POPE:POPG  EMD-49382  PDB 9NGC | ELIC5 with propylamine facing ECD inwards in liposomes with 2:1:1 POPC:POPE:POPG  EMD-49385  PDB 9NGI | ELIC with propylamine in spNW25 nanodiscs with 2:1:1 POPC:POPE:POPG  EMD-49400  PDB 9NH4 |
| --- | --- | --- | --- |
| **Data collection and processing** | | | |
| Magnification | 75000 | 75000 | 120000 |
| Voltage (kV) | 300 | 300 | 200 |
| Electron exposure (e-/ Å^2^) | 56.9 | 56.9 | 46.6 |
| Defocus range (µm) | -1 to -2.4 | -1 to -2.4 | -0.8 to -2.4 |
| Pixel size (Å) | 0.868 | 0.868 | 1.184 |
| Symmetry imposed | C5 | C5 | C5 |
| Initial particle images (no) | 422,344 | 422,344 | 1,031,340 |
| Final particle images (no) | 120,578 | 7,244 | 111,244 |
| Map resolution (Å) | 2.8 | 4.3 | 3.9 |
| FSC threshold | 0.143 | 0.143 | 0.143 |
| **Refinement** | | | |
| Initial model used | PDB 8VUW | PDB 8VUW | PDB 8F34 |
| Model resolution (Å) | 3.1 | 4.1 | 4.2 |
| FSC threshold | 0.5 | 0.5 | 0.5 |
| Map sharpening B factor (Å^2^) | -121.1 | -156.539 | -258.138 |
| **Model composition** | | | |
| Non-hydrogen atoms | 12722 | 12729 | 11320 |
| Protein Residues | 1550 | 1550 | 1375 |
| Ligands | 5 | 5 | 5 |
| **B factors (Å^2^) (0.5)** | | | |
| Protein | 78.30 | 139.9 | 32.33 |
| Ligand | 59.82 | 93.71 | 16.82 |
| **R.m.s. deviations** | | | |
| Bond lengths (Å) | 0.005 | 0.006 | 0.006 |
| Bond angles (°) | 1.083 | 1.189 | 1.169 |
| **Validation** | | | |
| MolProbity Score | 1.22 | 1.94 | 2.29 |
| Clashscore | 2.33 | 11.19 | 20.80 |
| Poor rotamers (%) | 0 | 0 | 0 |
| **Ramachandran plot** | | | |
| Favored (%) | 96.75 | 94.48 | 92.31 |
| Allowed (%) | 3.25 | 5.52 | 7.69 |
| Disallowed (%) | 0 | 0 | 0 |
