# Supplementary material for: Cryo-EM structures of a pentameric ligand-gated ion channel in liposomes
Source: eLife. 2025 Jul 16;14:RP106728. doi: 10.7554/eLife.106728 (PMC12266721; doi:10.7554/eLife.106728)
Supplement: Supplementary file 2. — The measurements show changes in the height of each transmembrane helix along the pore axis. [file elife-106728-supp2.docx]

|  | **M1 (W204 to L225)** | **M2 (F228 to T249)** | **M3 (V261 to F282)** |
| --- | --- | --- | --- |
| **WT ELIC with agonist in liposomes** | 32.3 Å | 32.5 Å | 31.0 Å |
| **WT ELIC with agonist in spNW25** | 30.5 Å | 31.9 Å | 30.2 Å |
| **WT ELIC with agonist in spMSP1D1 (PDB 8F34)** | 32.0 Å | 32.2 Å | 30.6 Å |
