# Supplementary material for: Cryo-EM structures of a pentameric ligand-gated ion channel in liposomes
Source: eLife. 2025 Jul 16;14:RP106728. doi: 10.7554/eLife.106728 (PMC12266721; doi:10.7554/eLife.106728)
Supplement: Supplementary file 3. — The area at the interface between subunits was determined using the PDBePISA server (Krissinel and Henrick, 2007), and the buried surface area between M4 and the rest of the ELIC protein was determined using ChimeraX1.6.1. [file elife-106728-supp3.docx]

|  | **WT ELIC with agonist** | | |
| --- | --- | --- | --- |
|  | **MSP1E3D1 (PDB 8D66)** | **spMSP1D1 (PDB 8F34)** | **Liposome (PDB 9NGQ)** |
| **TMD Interface (area)** | 1176.7 Å | 1176.8 Å | 1096.3 Å |
| **Buried surface area between M4 and the rest of ELIC** | 771.7 Å | 757.7 Å | 732.7 Å |
| **Total buried surface area** | 24864.7 Å | 25183.4 Å | 23947.3 Å |
|  | | | |
|  | **ELIC5 with agonist** | | |
|  | **MSP1E3D1 (PDB 8VUW)** | **spNW15 (PDB 8TWV)** | **Liposome (PDB 9NGC)** |
| **TMD Interface (area)** | 1171.1 Å | 1119.0 Å | 1091.7 Å |
| **Buried surface area between M4 and the rest of ELIC** | 842.0 Å | 986.2 Å | 870.3 Å |
| **Total buried surface area** | 25312.0 Å | 25065.1 Å | 24166.5 Å |
